# DNA Barcoding Survey of Anurans across the Eastern Cordillera of Colombia and the Impact of the Andes on Cryptic Diversity

**DOI:** 10.1371/journal.pone.0127312

**Published:** 2015-05-22

**Authors:** Carlos E. Guarnizo, Andrea Paz, Astrid Muñoz-Ortiz, Sandra V. Flechas, Javier Méndez-Narváez, Andrew J. Crawford

**Affiliations:** 1 Departamento de Zoologia, Universidade de Brasília, 70910–900, Brasília, DF, Brazil; 2 Department of Biological Sciences, Universidad de los Andes, A.A. 4976, Bogotá, Colombia; 3 Department of Basic Sciences, Universidad de la Salle, Bogotá, Colombia; 4 Smithsonian Tropical Research Institute, Apartado, 0843–03092, Panamá, Republic of Panama; Smithsonian Conservation Biology Institute, UNITED STATES

## Abstract

Colombia hosts the second highest amphibian species diversity on Earth, yet its fauna remains poorly studied, especially using molecular genetic techniques. We present the results of the first wide-scale DNA barcoding survey of anurans of Colombia, focusing on a transect across the Eastern Cordillera. We surveyed 10 sites between the Magdalena Valley to the west and the eastern foothills of the Eastern Cordillera, sequencing portions of the mitochondrial 16S ribosomal RNA and cytochrome oxidase subunit 1 (CO1) genes for 235 individuals from 52 nominal species. We applied two barcode algorithms, Automatic Barcode Gap Discovery and Refined Single Linkage Analysis, to estimate the number of clusters or “unconfirmed candidate species” supported by DNA barcode data. Our survey included ~7% of the anuran species known from Colombia. While barcoding algorithms differed slightly in the number of clusters identified, between three and ten nominal species may be obscuring candidate species (in some cases, more than one cryptic species per nominal species). Our data suggest that the high elevations of the Eastern Cordillera and the low elevations of the Chicamocha canyon acted as geographic barriers in at least seven nominal species, promoting strong genetic divergences between populations associated with the Eastern Cordillera.

## Introduction

The Northern Andes of South America have the highest diversity of species on Earth per unit of surface area and host multiple hotspots of species richness and endemism for plants and vertebrates [1–3]. In spite of its megadiversity, the number of described species in the Andes appears to be considerably underestimated [4–6]. The limited resources to study the Andean biodiversity is unfortunate since the tropical Andes is among the most threatened biomes in the world due to emergent pathogens [7], habitat deterioration [8], and global climate change [9–11]. Some evidence suggests that a major global wave of extinction has already begun [12,13], implying that much of the Andean undiscovered diversity may disappear before it is described by science.

Among vertebrates, tropical frogs have the highest species discovery rate over recent decades [14–20]. Intrinsic characteristics of anurans, such as their permeable skin and small body size [21–23], combined with their low dispersal abilities [24,25], and for some species, niche conservatism [1,26], promote geographic isolation and speciation, especially in heterogeneous topographies at middle elevations [1,27]. Unfortunately, tropical frogs are also among the most threatened vertebrates of the tropics [28,29]. Some authors suggest that the same attributes that make amphibians so diverse could also make them particularly susceptible to human-induced threats such as habitat fragmentation, climate change, and pathogens [30,31]. For these reasons, amphibian biodiversity surveys should be a priority in the Andes. The fast and accurate description of novel diversity of amphibians in tropical mountains would help us not only to understand why the Andes are so diverse, but also to design better conservation strategies to reduce potential negative impacts of further habitat degradation.

In Colombia, the Andes are divided into three cordilleras. Among these, the Eastern Cordillera is the widest in terms of area, and harbors higher species richness of vertebrates compared with the other two cordilleras (excluding the coastal Pacific slope of the Western Cordillera, also known as biogeographic Chocó [32]). The Eastern Cordillera lies between a humid lowland savanna (with Amazonian influences) drained by the Orinoco and Amazon rivers on the east side and the relatively arid Magdalena Valley on the west side. The two slopes of the Eastern Andes are separated by the Eastern Andes ridge, an uninterrupted ridge at ≥ 2500 m that extends for 600 km, and are, therefore, potentially under different environmental conditions that may promote alternative local adaptations. The species diversity of the eastern slope of the Eastern Cordillera is higher for organisms such as butterflies, frogs, birds, rodents, and bats [32], perhaps reflecting the fact that, A) the eastern slope receives high humidity from the north-eastern trade winds, promoting an Amazonian-influenced fauna in its foothills, in contrast to the relatively arid western foothills [32], and B) the eastern slope faces the Colombian and Venezuelan savannas in the north and the Amazonian lowlands in the south, facilitating biological exchange between the Eastern Cordillera and these contrasting regions.

Several authors have provided important insights on the processes that may explain the tremendous diversity of vertebrates of the Eastern Cordillera of Colombia [32–35]. These studies agree that the dynamic and recent uplift history of the Eastern Cordillera [36] makes geological history and topography a strong candidate to explain current diversity patterns. Lynch et al. [37], for instance, suggested that the recently uplifted Andes promoted diversification in two different ways: a) by fragmenting lowland populations on either side of the Eastern Andes ridge, and b) by fragmenting populations by means of nonsynchronous uplifting of Andean blocks that generate topographic complexity restricting gene flow. These authors observed that several closely related frog species occur allopatrically in similar elevational bands, supporting the idea that high summits and low valleys isolate populations and promote diversification. Another perspective suggests that more recent climatic cycles during the Quaternary had a profound effect of the demographic patterns of Andean species, alternately promoting or restricting migration during the glacial and interglacial periods [35].

While the fundamental insights above were reached through abundant morphology-based taxonomic studies on the vertebrates of Colombia’s Eastern Cordillera, the region is remarkably unexplored in terms of molecular systematic and phylogeographic analyses. While the number of molecular studies of vertebrates in this region has been increasing in recent years [38–45], the total number of studies is still very low relative to the remarkable species diversity. Tropical biodiversity studies at the molecular genetic level tend to reveal many undescribed or ‘cryptic’ species because speciation is not always accompanied by obvious morphological evolution [46]. Thus, we expect that genetic surveys of the Eastern Cordillera of Colombia should reveal further cryptic diversity, and therefore, novel insights on the patterns and processes associated with diversification.

DNA barcoding is a methodology that can be used for “specimen identification”, i.e., to assign unidentified specimens to named species, or “species discovery”, i.e., to assign novel specimens to unnamed genealogical clusters that may correspond to species [47]. In general, barcode algorithms for specimen identification compare individual DNA sequences against a reference library of homologous sequences from known samples whose identification is supported by curated voucher specimens. If genetic distances between the unknown sample and any known species in the reference library are smaller than a pre-established threshold, the unknown specimen likely corresponds to the closest species in the reference collection. Alternatively, if genetic distances are larger than the threshold, the unknown specimen may correspond to a species not in the reference library or possibly to an as yet undescribed new species [48]. This threshold is known as the barcode gap [49]. This technique is controversial because thresholds may vary among taxonomic groups, they may not be agreed upon by taxonomist, or they may not exist at all, i.e., there may be substantial overlap between intra- and interspecific pairwise genetic distances within one taxonomic group [49,50]. In response to this potential drawback several algorithms have been designed to try to optimize the threshold selection, or compare multiple thresholds in one barcode analysis [51–53].

DNA barcode analyses for species discovery are proficient at organizing unknown specimens into groups or clusters based on genetic distance or genealogical information. No algorithm, however, can confirm if these divergent clusters are indeed species, given that DNA sequence data from a single gene may not provide enough information for species delimitation [54]. Thus, while DNA barcodes cannot identify new species, they can help greatly in flagging unusual specimens that merit more careful revision using taxonomic characters appropriate for the group in question [55]. Under a program of integrative taxonomy, for example, candidate species are lineages that show divergent DNA sequence data as well as distinctiveness in one other source of character information, such as morphology, while ‘unconfirmed candidate species’ is a provisional designation based on DNA data or alternative characters [55].

Another potential drawback that might appear in DNA barcode analyses is the lack of consideration for geographic variation [53,56]. If sampling individuals from single localities, the potentially increasing variation in genetic distance over increasing geographical distance within species [57] may not be considered and the distinctiveness of species barcodes may be overestimated [53]. In other words, new sequences that originate from outside of the sampled range are easily misinterpreted as coming from other species [58]. Recent studies have shown, however, that adding samples from a greater proportion of the species range, even the addition of a single sample from a different population, leads to a higher number of accurate DNA barcode identifications [51,58].

We performed the first DNA barcoding survey of anurans of Colombia that includes multiple species, genera and families, along with multiple localities per species (when possible), focusing on a transect passing through the Eastern Cordillera of the Colombian Andes, starting in the foothills of the savannah, or Llanos region, in the east and ending in the lowlands of the Magdalena Valley on the western side. Our main questions were: A) compared with external morphology, what is the performance of DNA barcoding of anurans in terms of specimen identification and species discovery in the Eastern Cordillera of the Colombian Andes? And B) is there genetic evidence that the Eastern Cordillera ridge has acted as a geographic barrier separating eastern and western populations? Our study provides the first molecular sampling and analysis of anurans along a geographical transect across both slopes of the eastern Cordillera of Colombia. Our results shed light on the effectiveness of the DNA barcode methodology in accomplishing in a short period of time specimen identification and species discovery in a high-diversity tropical region.

## Methods

### Sampling

**Fig 1:**
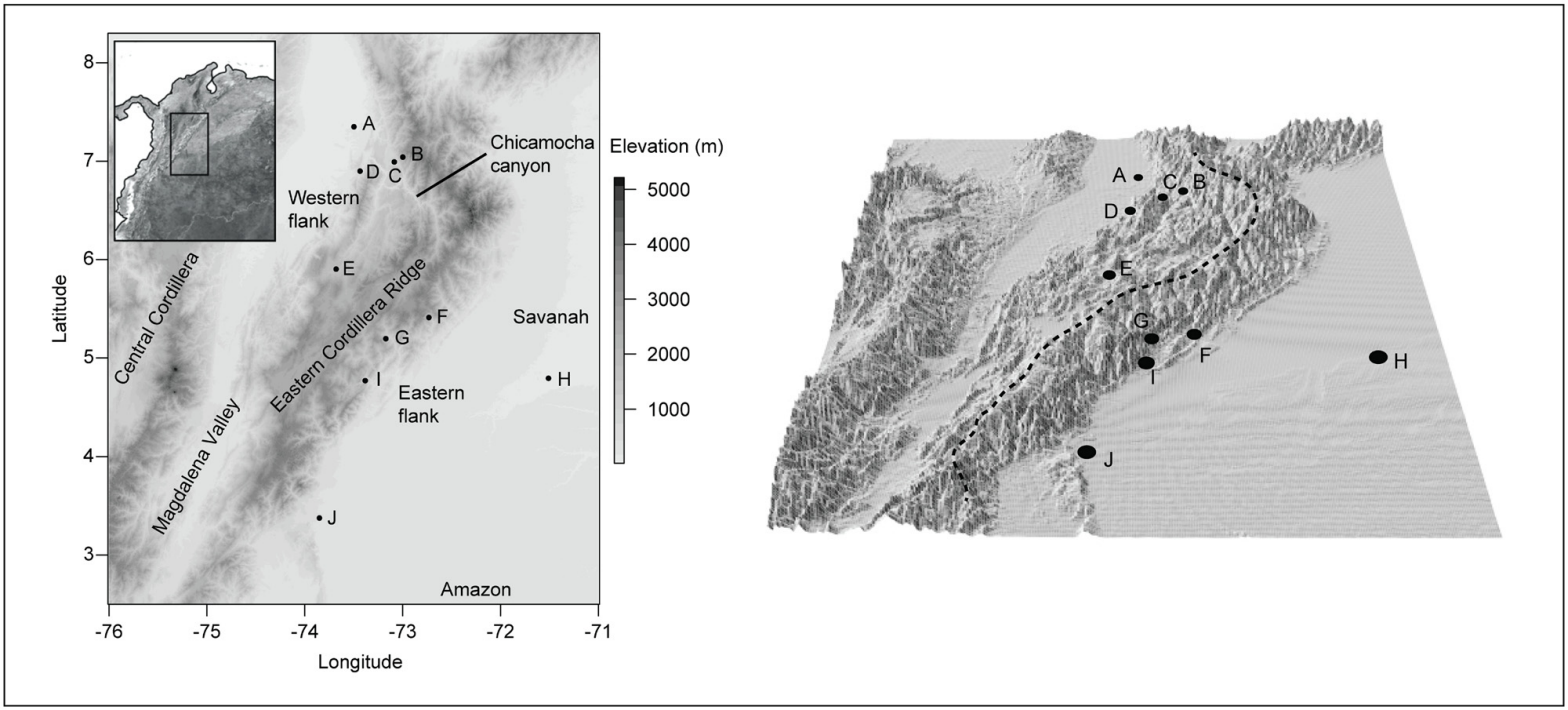
Geographic distribution of the ten localities surveyed for anurans. Left: Map codes refer to the localities listed in Table 1. Right: Three-dimensional map with vertical exaggeration (the vertical scale is larger than the horizontal scale) that displays the topographic complexity of the terrain. Black dots and numbers indicate the same localities shown on the left.

Specimens were sampled from 10 sites along an elevation transect across the Eastern Cordillera of Colombia (Fig 1, Table 1). Between 3 and 4 people worked during each fieldtrip to each locality, providing a mean sampling effort of 80 person-hours per site. Sampled individuals were preliminary identified and allocated to nominal species using external morphology. We collected a maximum of five individuals of the same nominal species at each locality. Sampled individuals were euthanized by topical application of benzocaine gel (i.e., tooth ache medicine). Tissue samples for DNA barcode analyses were obtained from the liver or skeletal muscle tissue and stored in an NaCl-saturated 0.25 M EDTA buffer containing 20% DMSO [59]. Vouchers were fixed in 10% formalin or 85% ethanol, stored in 70% ethanol and then deposited in the *Museo de Historia Natural ANDES* at the Universidad de los Andes. The species names, field collection numbers, museum numbers, localities, Barcode of Life Data Systems (BoLD) Process ID [60] for each specimen, and GenBank accession numbers for each sequence used in the present study are provided in the S1 Table.

**Table 1:** Description of localities and individuals used in the barcode analyses. For map codes see Fig 1. Localities are ordered from north to south.

Locality	Map code	Lat N, Long W	Elevation (m)	Slope of the Eastern Cordillera	Number of individuals collected	Number of individuals sequenced (gene)
Sabana de Torres	A	7.3496, -73.4981	159	Western	21	21 (16S), 18 (CO1)
El Rasgón	B	7.0416, -72.9588	2849	Western	10	10 (16S), 10 (CO1)
Piedecuesta	C	6.9923, -73.0528	1000	Western	6	6 (16S), 6 (CO1)
San Vicente	D	6.8988, -73.4305	474	Western	23	23 (16S), 21 (CO1)
Puente Nacional	E	5.9028, -73.6949	1702	Western	24	24 (16S), 22 (CO1)
Pajarito	F	5.4108, -72.6710	2351	Eastern	13	13 (16S), 13 (CO1)
Miraflores	G	5.1953, -73.1462	1532	Eastern	27	27 (16S), 25 (CO1)
Orocué	H	4.7969, -71.3512	134	Eastern	15	15 (16S), 14 (CO1)
Sabanalarga	I	4.7729, -73.3737	313	Eastern	51	50 (16S), 41 (CO1)
San Juan de Arama	J	3.3765, -73.8790	448	Eastern	47	47 (16S), 40 (CO1)

### PCR and sequencing methods

Genomic DNA was extracted using the DNeasy Blood & Tissue kit (Qiagen, Valencia, CA, USA) following the manufacturer’s protocol. We amplified two mitochondrial gene fragments, including the 5’ end of the faster evolving cytochrome oxidase subunit I (CO1) gene, also known as the DNA Barcode of Life for animals [61], and the more slowly evolving ribosomal 16S gene fragment which still boasts greater representation of amphibians in GenBank [62]. Two primer pairs were used to amplify and Sanger-sequence the CO1-5’ gene: dgLCO1490 (5’–GGTCAACAAATCATAAAGAYATYGG–3’) plus dgHCO2198 (5’–TAAACTTCAGGGTGACCAAARAAYCA–3’) [63], and Chmf4 (5’–TYTCWACWAAYCAYAAAGAYATCGG–3’) plus Chmr4 (5’—ACYTCRGGRTGRCCRAARAATCA–3’) [64]. The 16S gene fragment was amplified and sequenced with the primers 16Sar (5'–CGCCTGTTTATCAAAAACAT–3') and 16Sbr (5'–CCGGTCTGAACTCAGATCACGT–3') and standard reaction conditions [65]. PCR products were cleaned using Exonuclease I and Shrimp Alkaline Phosphatase enzymes [66]. Both genes were sequenced bi-directionally to confirm base calls. Assembly and editing of chromatograms were performed with SEQUENCHER software version 4.7 (GeneCodes Corp., Ann Arbor, MI, USA). The sequences of each gene were aligned independently using MUSCLE [67] and reviewed by eye. Our alignments are available from the Dryad data repository: doi:10.5061/dryad.k4q1q.

### DNA barcoding methods

Species identity and species discovery were implemented using two algorithms designed to statistically detect barcode gaps and identify distinct clusters of DNA sequences. First, we used the Automated Barcode Gap Discovery or ABGD method (available at http://wwwabi.snv.jussieu.fr/public/abgd/). ABGD first estimates the distribution of pairwise genetic distances between the aligned sequences and then it statistically infers multiple potential barcode gaps as minima in the distribution of pairwise distances, thereby partitioning the sequences such that the distance between two sequences taken from distinct clusters will be larger than the barcode gap [52]. The software also recursively applies this procedure to the previously obtained groups of sequences to get finer partitions. Since the analysis identifies similar DNA sequences first, without removing gapped sites in the alignment, ABGD (and RESL, below) is not affected by gaps caused by the inclusion of divergent samples such as additional taxonomic genera or families. The ABGD algorithm has been shown to provide good performance in terms of species identification compared with other DNA barcode algorithms [51]. The CO1, 16S, and concatenated alignments were processed in ABGD using the complete sequence data, the Kimura two-parameter (K2P) nucleotide substitution model [68], and the following settings: prior for the maximum value of intraspecific divergence between 0.001 and 0.1, 10 recursive steps within the primary partitions defined by the first estimated gap, and a gap width of 1.0. K2P is the standard model of DNA substitution for barcode studies, and performs as well as more complex models in identifying specimens [69].

In order to contrast the results obtained by the ABGD algorithm we used the Barcode Index Number discordance analysis available in BoLD [70], a public DNA sequence database devoted to aiding in the acquisition, storage, analysis, and publication of DNA barcode records including chromatograms, images, collection data, and additional DNA sequences (http://www.boldsystems.org). The method uses a Refined Single Linkage (RESL) Analysis algorithm, which follows two main steps: First, it employs single linkage clustering as a tool for the preliminary assignment of the CO1 sequences to a cluster (using genetic distances between every pair of sequences), and second, it uses Markov clustering, a method that groups together CO1 sequences with high sequence similarity and connectivity, and separates those with lower similarity and sparse connectivity. Such connectivity is explored through random walks of the genetic distance similarity network [70]. Finally, RESL defines the boundaries of each cluster selected by generating clusters using a range of values for the inflation parameter in the Markov clustering and then selects that which maximizes the Silhouette Index [65]. Since the RESL algorithm was designed to deal with large quantities of sequences, it does not need prior information on the barcode gap, as with ABGD.

The RESL assignment is only performed for specimens with CO1 sequences of more than 300 base pairs (bp), and thus we applied this method to 213 specimens (see below). In order to be able to compare ABGD and RESL in terms of the number of clusters inferred, we performed an additional analysis using the same CO1 alignment in both algorithms. We did not included more algorithms in our analysis since our main focus was to contrast how different clusters of individuals are formed using either external morphology or DNA, rather than comparing the performance of several different DNA barcode algorithms, as previous studies have already done [51,71–73].

In addition to the distance-based analyses mentioned above, we also evaluated a character-based phylogenetic approach. We estimated maximum likelihood (ML) gene genealogies for each gene and the combined dataset using RAxML-VI-HPC [74] with a GTRGAMMA model of evolution. We conducted 100 independent ML tree searches and used the—f a option implementing 1000 rapid bootstrap replicates. Our intention in estimating the ML tree was to obtain the bootstrap support for derived nodes that may represent species, rather than attempting to estimate phylogenetic relationships among genera or families.

### Colombian Eastern Andes as a geographic barrier

To test if the Eastern Andes ridge affected the local genetic differentiation of the species sampled across both sides of the Eastern Cordillera we used the program LocalDiff [75]. This program uses a Bayesian approach to characterize non-stationary patterns of isolation by distance, in other words, when genetic differentiation between individuals increases at different rates in different regions of the habitat [75]. Non-stationary patterns of isolation by distance may arise in the presence of barriers to gene flow because genetic differentiation accumulates faster with distance around the barrier [75].

Since local genetic differentiation (among populations over small geographical scales) should be larger in regions of abrupt genetic changes, we estimated a similarity matrix 1 –(observed genetic distance/maximum genetic distance) as a measure of intraspecific local differentiation. Genetic distances were normalized dividing them by the maximum genetic distance found within each lineage, so they would rank between 0 (the site with the lowest local differentiation) and 1 (the site with the highest local differentiation), facilitating its comparison with other studies. Local genetic differentiation was estimated with kriging [76], a method that interpolates genetic distances in unsampled neighboring sites based on genetic distances measured at the sampling sites, such that local genetic differentiation can be estimated continuously across space. The interpolation is dependent on a weighted average of the values measured at the sampling sites, and the weights rely on a parametric function that provides the decay of the similarity matrix with geographic distance. The output of the program LocalDiff generates a minimum convex polygon that encompasses all sampling sites within a species, where warmer colors represent areas with stronger local genetic differentiation. The local differentiation at each sampled location corresponds to the average (over neighbors) pairwise genetic distance between the individual at the sampled location and a putative neighboring site at a pre-specified geographic distance [76].

Genetic distances between all 16S haplotype pairs were estimated assuming the K2P model in MEGA 4.0 [77], using the pairwise-deletion option. We used the 16S dataset because, as opposed to CO1, 16S data were obtained from all geographic sites. In LocalDiff we implemented the following parameters: 2 fictional neighboring populations in the vicinity of each sampled site, and a geographic distance between the neighbors and the sampling sites of 0.1 km. LocalDiff measures were averaged over 1000 replicates.

Because LocalDiff is an extension of isolation by distance [57], we checked if population differentiation indeed increased with increasing geographical distance by estimating the relationship between geographic and genetic distances with multiple matrix regression with randomization (MMRR) [78], which tests the significance of a simple or multiple regression using a randomized permutation procedure to account for the potential non-independence among samples. Geographic distances were estimated with Geographic Distance Matrix Generator [79]. We performed the MMRR method using the R function from Ian Wang [78] with 10,000 permutations.

### Ethics Statement

Procedures for capture and handling of live animals in the field were approved by the Colombian Ministry of the Environment, under research and collecting permit N°15 and access to genetic resources permit N°44. None of the species collected for this study is listed in the Convention on International Trade in Endangered Species of Wild Fauna and Flora—CITES (www.cites.org). The frogs were collected on private property, and permission was received from landowners prior to sampling.

## Results

We surveyed ten localities (Table 1) and collected 232 individual frogs representing eight taxonomic families, 25 genera, and 52 nominal species (Table 2). The localities on the eastern slope of the Eastern Cordillera all showed greater species richness than sites on the western slope. 52% of nominal species were collected on the eastern slope, 36% on the western slope, and 11% were found on both sides of the Eastern Cordillera (Table 2).

**Table 2:** Description of the nominal species identified a priori with external morphology. Species are organized alphabetically by family and then by genera.

Nominal species	Family	Number of individuals within species	Species found in how many localities?	Flank of the Eastern Cordillera
*Allobates niputidea*	Aromobatidae	1	1	Western
*Allobates ranoides*	Aromobatidae	2	1	Eastern
*Rheobates palmatus*	Aromobatidae	9	3	Both
*Rhinella humboldti*	Bufonidae	4	2	Eastern
*Rhinella margaritifera*	Bufonidae	5	2	Eastern
*Rhinella marina*	Bufonidae	10	5	Both
*Espadarana andina*	Centrolenidae	2	1	Western
*Hyalinobatrachium esmeralda*	Centrolenidae	1	1	Eastern
*Rulyrana flavopunctata*	Centrolenidae	1	1	Eastern
*Craugastor longirostris*	Craugastoridae	2	1	Western
*Pristimantis carranguerorum*	Craugastoridae	1	1	Eastern
*Pristimantis douglasi*	Craugastoridae	3	1	Western
*Pristimantis frater*	Craugastoridae	6	2	Easten
*Pristimantis lutitus*	Craugastoridae	1	1	Western
*Pristimantis miyatai*	Craugastoridae	7	2	Western
*Pristimantis savagei*	Craugastoridae	6	2	Eastern
*Pristimantis taeniatus*	Craugastoridae	3	1	Eastern
*Pristimantis vilarsi*	Craugastoridae	6	1	Eastern
*Dendrobates truncatus*	Dendrobatidae	3	1	Western
*Agalychnis terranova*	Hylidae	2	1	Western
*Dendropsophus ebraccatus*	Hylidae	2	1	Western
*Dendropsophus mathiassoni*	Hylidae	13	3	Eastern
*Dendropsophus microcephalus*	Hylidae	4	2	Western
*Dendropsophus* cf. *stingi*	Hylidae	10	3	Eastern
*Dendropsophus subocularis*	Hylidae	3	1	Western
*Hyloscirtus* cf. *phyllognathus*	Hylidae	1	1	Eastern
*Hypsiboas boans*	Hylidae	1	1	Eastern
*Hypsiboas crepitans*	Hylidae	14	5	Both
*Hypsiboas lanciformis*	Hylidae	4	1	Eastern
*Hypsiboas pugnax*	Hylidae	7	2	Western
*Hypsiboas punctatus*	Hylidae	6	2	Eastern
*Phyllomedusa hypochondrialis*	Hylidae	4	2	Eastern
*Pseudis paradoxa*	Hylidae	1	1	Eastern
*Scinax* cf. *kennedyi*	Hylidae	5	2	Eastern
*Scinax rostratus*	Hylidae	7	3	Both
*Scinax ruber*	Hylidae	10	4	Both
*Scinax wandae*	Hylidae	7	2	Eastern
*Smilisca phaeota*	Hylidae	1	1	Western
*Trachycephalus typhonius*	Hylidae	1	1	Eastern
*Adenomera andreae*	Leptodactylidae	3	1	Eastern
*Engystomops pustulosus*	Leptodactylidae	5	3	Western
*Leptodactylus colombiensis*	Leptodactylidae	7	3	Both
*Leptodactylus fuscus*	Leptodactylidae	6	4	Both
*Leptodactylus insularum*	Leptodactylidae	2	1	Western
*Leptodactylus knudseni*	Leptodactylidae	2	1	Eastern
*Leptodactylus lineatus*	Leptodactylidae	3	1	Eastern
*Leptodactylus mystaceus*	Leptodactylidae	1	1	Eastern
*Physalaemus fischeri*	Leptodactylidae	6	3	Eastern
*Pleurodema brachyops*	Leptodactylidae	1	1	Western
*Pseudopaludicola llanera*	Leptodactylidae	7	3	Eastern
*Pseudopaludicola pusilla*	Leptodactylidae	2	1	Western
*Elachistocleis ovalis*	Microhylidae	9	3	Eastern

**Fig 2:**
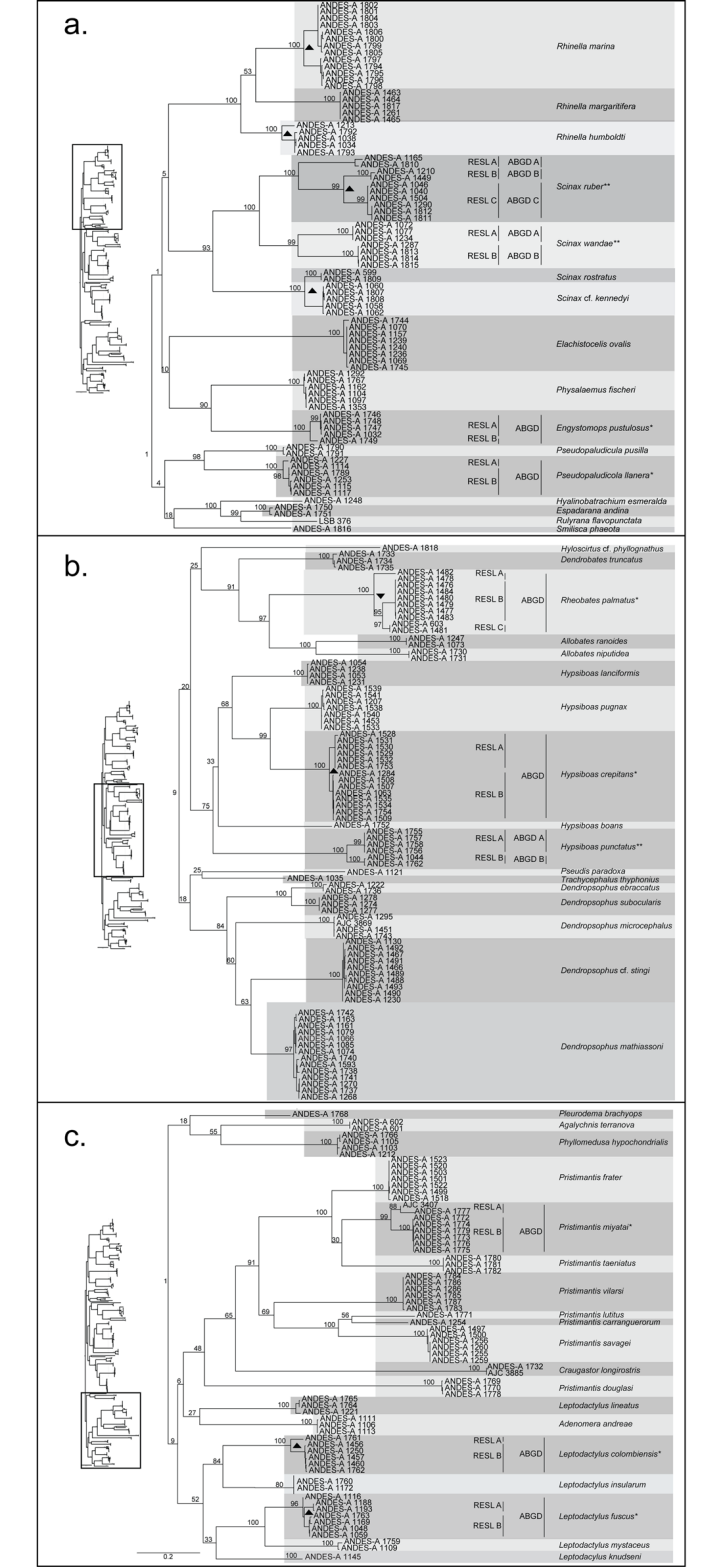
Maximum likelihood gene genealogy of the combined dataset (16S + CO1). Numbers on branches are bootstrap support values as estimated using the software RAxML. Horizontal grey boxes delineate the nominal species identified a priori. Nominal species with asterisks were divided into more than one cluster by at least one DNA barcoding algorithm. Vertical lines indicate the grouping of individuals by each algorithm, RESL and ABGD. Single asterisk (*) indicates nominal species that were split by RESL but not by ABGD. Double asterisks (**) indicate nominal species divided congruently by both algorithms. Nominal species without asterisks were grouped into a single cluster by both algorithms. Black triangles indicate clades geographically separated by the high-elevation ridge of the Eastern Cordillera. Black inverted triangles indicate clades geographically separated by the Chicamocha canyon.

We obtained DNA sequence data from the 16S ribosomal gene fragment for all 237 sampled individuals, consisting of 579 aligned bp, excluding gapped sites. We obtained CO1-5’ gene sequences for 213 individuals (658 bp, with no length variation among individuals). The discrepancy between the two genes in the number of individuals sequenced was due to problems with PCR amplification of the CO1-5’ fragment, despite using two sets of primer with numerous degenerate bases. The topologies of the character-based ML trees for CO1 and 16S agreed in terms of clade membership and support for terminal nodes, though not in terms of the relationships among basal groups. We therefore present here the ML tree based on the concatenated alignment as our best estimate, though again our goal was to identify nominal species and potential unconfirmed candidates species, not to resolve generic and family-level relationships (Fig 2). Bootstrap support for terminal nodes was high (>95%) but was low for basal nodes.

In terms of species delimitation, the ABGD algorithm produced a stable number of clusters (or “hypothetical species”) for each independent gene and the concatenated alignment across a wide number of intraspecific thresholds (S1 Fig). ABGD recovered 47 clusters based on 16S, 54 clusters based on CO1, and 56 clusters using the concatenated alignment. Given that both genes independently recovered congruent groups, from now on we discuss the groups formed in terms of the concatenated alignment (56 clusters), since it is supported by more data (when contrasting ABGD and RESL we refer to the CO1 alignment only, see below). For the concatenated matrix we chose 6% based on the observed ‘barcode gap’ in the bimodal distribution of pairwise genetic distances (S1 Fig). It is worth noticing that our concatenated alignment contained a small proportion of missing data, since 23 individuals were not sequenced for CO1. Therefore, the resolution might be lower in those clades with 16S sequence data only.

In three cases individuals identified as members of the same nominal species (given their strong morphological similarity) were genetically highly divergent and split into paired clusters by ABGD: *Scinax ruber* (3 clusters), *S*. *wandae* (two clusters), and *Hypsiboas punctatus* (two clusters). These three nominal species might, therefore, correspond to three named species plus four unnamed cryptic species according to the ABGD algorithm.

The RESL analysis employing only the CO1 data (Fig 2) recovered a total of 65 clusters (11 more than the ABGD algorithm using the same gene). Of these clusters, 10 records were singletons thus providing the first reports of these lineages in BoLD. In eight cases, individuals identified as members of the same nominal species were split by RESL into paired clusters: *S*. *wandae*, *Engystomops pustulosus*, *Pseudopaludicola llanera*, *Hypsiboas crepitans*, *H*. *punctatus*, *Pristimantis miyatai*, *Leptodactylus colombiensis*, and *L*. *fuscus*. Two additional nominal species were split into three clusters: *Scinax ruber* and *Rheobates palmatus*. These 10 nominal species might, therefore, contain an additional 11 unnamed cryptic species, according to the RESL algorithm.

The RESL algorithm appeared to have a slightly lower threshold separating CO1 haplotypes into different clusters compared with ABGD. For instance, the nominal species *E*. *pustulosus*, *P*. *llanera*, *H*. *crepitans*, and *L*. *fuscus* were grouped in a single cluster each by the ABGD algorithm, but split into two or three clusters each by the RESL algorithm, confirming that RESL is more sensitive and potentially more subject to false positives compared with ABGD. The remaining clusters were concordant between the two algorithms (85% of the nominal species). In summary, out of the original 52 nominal species for which we obtained CO1 data, the ABGD algorithm suggested 54 clusters based on a gap of 1.0, while the RESL algorithm increased to 65 the number of potential species.

We confirmed the prevalence of isolation by distance with the MMRR algorithm, finding significant (*P* < 0.05) associations between geographic and genetic distances in all seven nominal species sampled on multiple localities (*L*. *colombiensis*: r^2^ = 0.74, *P* = 0.0039; *H*. *crepitans*: r^2^ = 0.14, *P* = 0.0007; *L*. *fuscus*: r^2^ = 0.60, *P* = 0.0035; *R*. *palmatus*: r^2^ = 0.90, *P* = 0.0142; *S*. *ruber*: r^2^ = 0.40, *P* = 0.0002; *R*. *marina*: r^2^ = 0.23, *P* = 0.0007; and *R*. *humboldti*: r^2^ = 0.40, *P* = 0.03219).

**Fig 3:**
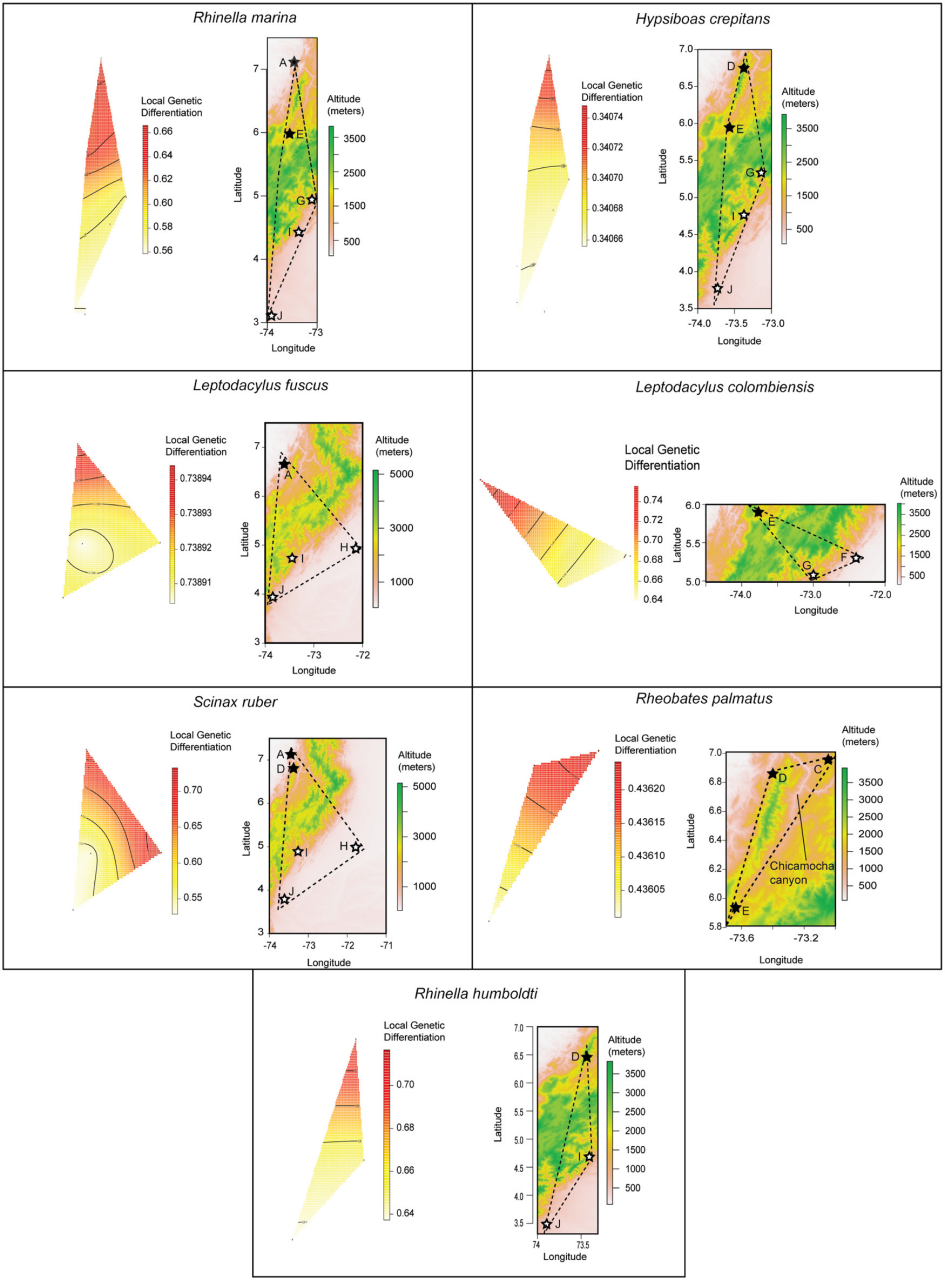
Map of local genetic differentiation in the seven nominal species sampled from both flanks of the Eastern Cordillera. For each species, the triangle on the left represents the minimum convex polygon that encompasses the sampling sites of each species. Warmer colors represent greater genetic distances between sampled sites and unsampled neighbors. The map on the right represents topography, with the dotted line indicated the spatial location of the polygon on the left. Black stars are western flank localities and white stars are eastern flank localities. Numbers correspond to map codes in Fig 1 and Table 1. Local genetic differentiation corresponds to one minus the expected correlation between sampled sites and neighboring unsampled sites located at 0.1 km.

The majority of species that were sampled on both sides of the Eastern Cordillera ridge (*R*. *marina*, *L*. *fuscus*, *L*. *colombiensis*, *H*. *crepitans*, and, *R*. *humboldti*) displayed a steep gradient in the local genetic differentiation (the genetic distance per unit of geographic distance) that increased parallel to the ridge (Fig 3). In these species, the more abrupt change between low local genetic differentiation (colder colors) and high local genetic differentiation (warmer colors) occurs near-by the geographic area that corresponds to the ridge (Fig 3), suggesting that a) the Eastern Cordillera ridge is acting as a barrier separating western flank and eastern flank sites, and b) that within these species, local genetic differentiation is higher in the western slope of the Eastern Cordillera. Even though we did not sample *R*. *palmatus* on both sides of the ridge, we found a steep change in local genetic differentiation in the area corresponding the Chicamocha canyon, a low elevation valley that reaches depths of 400 m (Fig 3). *Scinax ruber*, which had strongly divergent samples from a far eastern site (Orocué) in the Llanos east of the Cordillera was the only species showing a local genetic differentiation gradient perpendicular to the ridge. We were not able to test local genetic differentiation in the other nominal species given that these were collected in just one or two localities or the barcode algorithms divided them into different clusters.

## Discussion

### Species identification and discovery across the Eastern Cordillera

Our DNA barcoding survey of 52 nominal species corresponds to 7.3% of all anuran species of Colombia (of a total of 710 anurans recorded as of October 2014 [80]). In terms of species identification, external morphology revealed fewer entities than DNA barcoding, not surprisingly, with 52 nominal species identified with morphology versus 56 and 65 clusters identified with molecular data (ABGD and RESL, respectively).

The two DNA-based algorithms were somewhat dissimilar in terms of the agreement between the nominal species and clusters. ABGD revealed that only three of the nominal species identified *a priori* contained cryptic lineages, while in RESL, this number increased to ten. We suggest, then, that the ABGD algorithm would be more appropriate for identifying unconfirmed candidate species, as it is more conservative and groups haplotypes independently of their taxonomy. The RESL algorithm, on the other hand, would be preferable for species identification, since it is less conservative and compares the DNA barcodes against a reference database of identified samples, and thereby suggests names for the unknown sequences. Of course, any species identification method is only as reliable as the database upon which it is based [60], in this case BoLD.

Regarding species discovery, we found that the nominal species *E*. *pustulosus*, *P*. *llanera*, *H*. *crepitans*, *L*. *fuscus*, *R*. *palmatus*, *P*. *miyatai*, and *L*. *colombiensis*, were split into two or more groups by the less conservative RESL algorithm. Since this algorithm requires low genetic divergence to separate entities, these seven species might correspond to either highly divergent conspecific lineages, or unconfirmed candidate species separated by narrow genetic divergences. Amphibian species frequently show relative morphological stasis despite strong genetic divergences (well above the proposed thresholds to identify species using DNA barcode markers) [18,62]. Moreover, convergence and parallelism are often found among even relatively closely related amphibians [81,82]. It is likely, therefore, that species diversity becomes masked with the exclusive use of morphological characters to describe diversity [83]. A barcode approach allows even the non-specialist to be able to flag genetically divergent groups whose taxonomic status require further expert investigation using independent data.

The more conservative ABGD identified three nominal species that we believe are highly likely to contain cryptic diversity given their very deep genetic divergences: *H*. *punctatus*, *S*. *wandae* and *S*. *ruber*. This is not surprising for *S*. *ruber*, which has been shown to contain substantial cryptic diversity in a recent study of populations in French Guyana [84]. As *H*. *punctatus*, *S*. *wandae* and *S*. *ruber* are geographically even more widespread across lowland sites that our sampling included, we suspect each of these nominal species to contain a multitude of cryptic lineages each, overlooked by previous studies based in morphology. A good example of the underestimation of diversity in lowland frog species is the case of *Dendropsophus minutus*, a widespread species in South America that displays 43 statistically supported deep mitochondrial lineages, many of which likely correspond to undescribed cryptic species [85]. Curiously, both algorithms missed the two divergent clades within *Rhinella marina* separated by the eastern Cordillera ridge. We explain this result by the fact that *R*. *marina* had several CO1 missing sequences, decreasing the overall resolution of this clade and the performance of both algorithms identifying groups. This observation highlights the relevance of CO1 (compared with 16S) detecting groups separated by narrow genetic divergences, and also, the possible role that missing data can have on the performance of genetic barcode algorithms.

It is clear that the DNA barcode technique outperformed our morphological identifications in terms of both accuracy and time. The question is what to do with the outcomes of barcode surveys. For example, we found three highly divergent groups within the nominal species *S*. *ruber*, while Fouquet et al. [84] found six. Our candidates and Fouquet’s candidates are different entities since they were sampled in distant geographic regions. One of the goals of rapid biodiversity description is to characterize communities and biogeographic regions, such as the tropical Andes, in short time spans. The use of standardized markers and data bases goes a long way towards facilitating comparisons among disparate studies, yet ascribing a formal name to all these divergent groups might still be difficult, especially if it required the international shipment of type specimens. Padial et al. [55] suggested a strategy that could speed up the characterization of biogeographic regions using barcoding techniques. Their approach proposes to designate the provisional label of ‘unconfirmed candidate species’ to groups of specimens within nominal species that show unusually large genetic distances. The lineage would then be identified through the combination of the Linnaean binomial species name of the most closely related nominal species, followed by the abbreviation "Ca" (for candidate), adding a numerical code indicating the particular candidate species group. This system potentially would help researchers to be aware of what particular candidate species have been found, avoiding repeating or ignoring genetic clusters that have been already published. Also, it may speed up the naming process by taxonomists, making more evident the groups that contain undescribed diversity. Similarly, the standardized barcode database, BoLD, scans all CO1 sequences in the database and labels each cluster identified by its RESL algorithm numerically, creating a Barcode Index Number [70].

### The Eastern Cordillera as a geographic barrier

DNA barcode analyses can go beyond describing biodiversity patterns by studying the processes that generate or erase such patterns [48,86]. In addition to characterizing the diversity of lineages in our survey, we also explored the role that the Eastern Cordillera may have played in increasing intraspecific divergence within nominal species sampled from both flanks. A map of local genetic differentiation showed that in five out of six species, the steepest change in genetic distances per unit of geographic distances was parallel and in close proximity to the Eastern Andes ridge. Genetic differentiation varies spatially due to several factors, including migration and other demographic processes [86], extinction and re-colonization of demes [87] and of course historical biogeographic processes [88], and teasing apart these factors is often challenging. The fact that the same pattern was found in five species of different taxonomic families, however, strongly suggests that local genetic differentiation was not the product of idiosyncratic processes, but the general effect of the Eastern Andes ridge reducing gene flow between populations across both sides of the ridge.

The observation of genetically divergent conspecific populations from opposite slopes of the Andes has been described in multiple anuran species including *R*. *marina* [89], *E*. *pustulosus* [90], *R*. *palmatus* [42,91], and *Dendropsophus labialis* [43], as well as in several species of Andean birds [92,93]. This evidence supports the fact that tropical lowland species (as opposed to temperate species) have strong difficulties crossing high elevation summits, a hypothesis formulated by Janzen almost 50 years ago [94], prior to the advent of DNA sequence analyses. Also, we confirmed the results of Muñoz-Ortiz et al. [91] finding that valleys are also important geographic features that restrict gene flow in mid-elevation species such as *R*. *palmatus*. Wier [95] suggested that Andean low valleys are more important promoting vicariance than Andean high summits, because the major uplift activity of the northern Andes was too recent for strong genetic divergences to build-up across such barriers, whereas low-elevation barriers have had more time to generate divergence. We found strong evidence, however, that both high elevation summits and low elevation valleys are a prevalent force isolating populations and promoting genetic divergence in Andean anurans. This result agrees with Lynch et al. [34], suggesting that the nonsynchronous uplift of the Eastern Cordillera generated a topographic complexity that likely fragmented populations and promoted diversification in the past. Certainly, the comparison of high and low elevations as geographic barriers should be explored in more detail in future studies including more species and additional Andean landscapes.

We hypothesize that the reason why five different species showed higher local genetic differentiation in the western flank of the Eastern Cordillera (relative to the eastern flank) is because it has a more complex topography and climate than the homogeneous Llanos in the eastern flank (Fig 3), and complex topographies and climates have been found to increase genetic divergence [27,96]. Curiously, our survey found both higher genetic differentiation among sites and lower species richness in the western flank. In other words, it appears that the western flank has higher beta diversity relative to the eastern flank, while the eastern flank has higher alpha diversity. We hypothesize that this observation is explained by the fact that the eastern flank localities contain multiple widely distributed species living in sympatry, due to the lack of geographic barriers in the flat *Llanos Orientales*, while in the western flank the complex topography and environmental heterogeneity of the Magdalena Valley isolates populations, increasing the diversity among sites.

Even though we focused our discussion on the effects of topography and climate fragmenting anuran populations, we cannot claim these are the only factors playing role in the diversification of the Eastern Cordillera. There are abundant studies that have shown how the idiosyncratic natural history of each species can be relevant explaining biological diversification. For example, small body sizes in the Malagasy mantellid frogs have been shown to limit the dispersal capabilities and to low physiological tolerances, causing strongly fragmented ranges [23]. Also, call variation (instead of landscape features) has been shown to promote genetic divergence and speciation in the lowland Amazonian frog, *Engystomops petersi* [97]. Even though we agree with Lynch et al. [34] that topography and climate are in general the most important factors explaining the current diversity of the Eastern Cordillera, it is fundamental to further explore how the natural history of Andean species promotes their diversification or extinction.

We found clear evidence for the key role of the Eastern Cordillera in creating genetic divergence at the nominally intraspecific level. More evidence, however, needs to be collected in order to determine not only how the Andes shape genetic variation, but also how it shapes the ecology and phenotypes of these species.

## Conclusions

Here we performed the first wide-scale DNA barcode survey of anurans in Colombia. We found that DNA barcoding is a method that outperformed our morphological identifications allowing us to complete the survey in a short period of time and to flag specimens from populations that require further taxonomic and ecological scrutiny. We also found, using a novel Bayesian kriging approach, that both summits and valleys of the Eastern Andes of Colombia are important geographic features promoting intraspecific genetic divergence across several frog species, and that the eastern and western flanks have contrasting diversity levels. It is somewhat accepted that tropical lowland species have rather wide geographic distributions, while montane species have very restricted ranges. We believe, however, that due to morphological stasis, a significant proportion of lowland species diversity has been underestimated by the predominant use of morphology in previous estimations [85,98]. It is quite possible that future genetic analyses will keep fragmenting the wide distributions of lowland species, which might change our current perception of tropical lowland vs. highland diversity patterns. We recommend that DNA barcoding surveys spend the additional effort, when possible, to collect multiple specimens from multiple localities that cover the geographic extent of each species in order to recover a better proportion of the intraspecific variability [99], which in turn allows for extended analyses of the genetic variation of the species found in the survey and their geographic patterns. The tropical Andes in general, and Colombia specifically, still represent a poorly known region in terms of its biodiversity. We encourage researchers to include DNA barcode analyses in addition to the fundamental morphological analyses in future surveys of biodiversity of the tropical Andes [98]

## Supporting Information

S1 Fig ABGD additional information. Left: Histogram showing the distribution of pairwise genetic distances (Kimura 2-parameter) among all samples using the combined dataset. The arrow indicates the threshold selected as separating within-species genetic variation and between-species genetic divergence. Right: Plot depicting how the number of clusters or hypothetical species recovered by the ABGD algorithm varies across increasing ‘prior intraspecific genetic divergences’ or thresholds. Recursive partitions are obtained by allowing the threshold to vary among species.
(PDF)

S1 Table Detailed information on the samples collected. Field number, museum code, nominal species designation, collecting locality, BoLD ProcessID, ABGD cluster membership, RESL cluster membership (BIN), and GenBank accession number for each sample included in this study. NA correspond to missing voucher specimens.
(PDF)
