# Supplementary material for: DNA Barcoding Survey of Anurans across the Eastern Cordillera of Colombia and the Impact of the Andes on Cryptic Diversity
Source: PLoS One. 2015 May 22;10(5):e0127312. doi: 10.1371/journal.pone.0127312 (PMC4441516; doi:10.1371/journal.pone.0127312)

S1 Fig. ABGD additional information

Left: Histogram showing the distribution of pairwise genetic distances (Kimura 2-parameter) among all samples using the combined dataset. The arrow indicates the threshold selected as separating within-species genetic variation and between-species genetic divergence. Right: Plot depicting how the number of clusters or hypothetical species recovered by the ABGD algorithm varies across increasing 'prior intraspecific genetic divergences' or thresholds. Recursive partitions are obtained by allowing the threshold to vary among species.

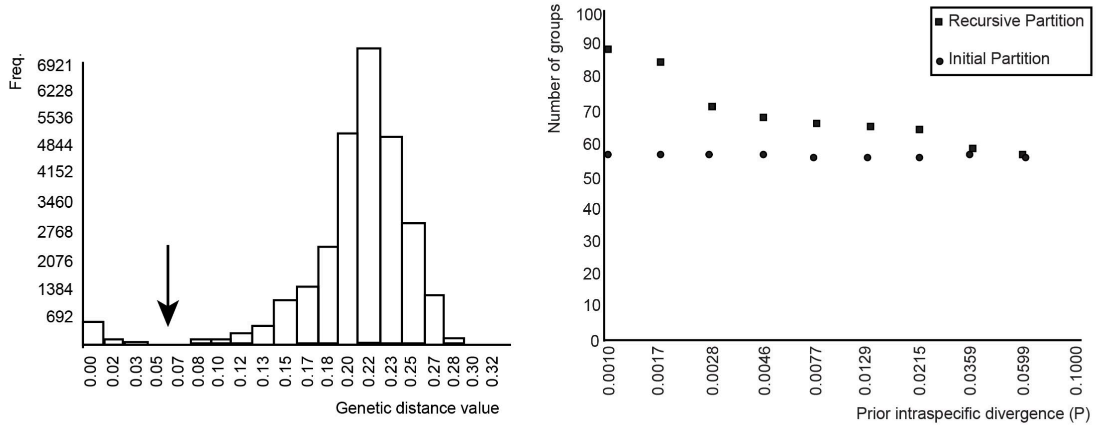

Supplement: S1 Fig — Left: Histogram showing the distribution of pairwise genetic distances (Kimura 2-parameter) among all samples using the combined dataset. The arrow indicates the threshold selected as separating within-species genetic variation and between-species genetic divergence. Right: Plot depicting how the number of clusters or hypothetical species recovered by the ABGD algorithm varies across increasing ‘prior intraspecific genetic divergences’ or thresholds. Recursive partitions are obtained by allowing the threshold to vary among species. (PDF) [file pone.0127312.s001.pdf]
