# Supplementary material for: DNA Barcoding Survey of Anurans across the Eastern Cordillera of Colombia and the Impact of the Andes on Cryptic Diversity
Source: PLoS One. 2015 May 22;10(5):e0127312. doi: 10.1371/journal.pone.0127312 (PMC4441516; doi:10.1371/journal.pone.0127312)
Supplement: S1 Table — Field number, museum code, nominal species designation, collecting locality, BoLD ProcessID, ABGD cluster membership, RESL cluster membership (BIN), and GenBank accession number for each sample included in this study. NA correspond to missing voucher specimens. (PDF) [file pone.0127312.s002.pdf]

S1 Table. Detailed information on the samples collected.

Field number, museum code, nominal species designation, collecting locality, BoLD ProcessID, ABGD cluster membership, RESL cluster membership (BIN), and GenBank accession number for each sample included in this study. NA correspond to missing voucher specimens.

Field number	Museum Number	Nominal species	Locality	Process BOLD	OTUs ABGD (16S + cox1)	OTUs RESL (cox1)	16S GenBank Accession #	CO1 GenBank Accession #
AJC 4091	ANDES-A 1111	*Adenomera andreae*	Sabanalarga, Casanare	BSECO042-11	53	BOLD:ABU5979	KP149345	KP149148
AJC 4093	ANDES-A 1106	*Adenomera andreae*	Sabanalarga, Casanare	BSECO044-11	53	BOLD:ABU5979	KP149454	KP149245
AJC 4094	ANDES-A 1113	*Adenomera andreae*	Sabanalarga, Casanare	BSECO045-11	53	BOLD:ABU5979	KP149377	KP149175
AJC 3510	ANDES-A 602	*Agalychnis terranova*	San Vicente, Santander	BSAMS118-12	31	BOLD:ACC1259	KP149386	KP149183
AJC 3515	ANDES-A 601	*Agalychnis terranova*	San Vicente, Santander	BSAMS120-12	31	BOLD:ACC1259	KP149341	KP149144
AJC 3897	ANDES-A 1730	*Allobates niputidea*	Sabana de Torres, Santander	BSECO324-11	44	Not sequenced for CO1	KP149362	Not sequenced for CO1
AJC 3900	ANDES-A 1731	*Allobates niputidea*	Sabana de Torres, Santander	BSECO327-11	44	BOLD:ABV7978	KP149439	KP149233
AJC 3383	ANDES-A 1073	*Allobates ranoides*	Sabanalarga, Casanare	BSECO100-11	14	BOLD:ABA1656	KJ130697	KJ130661
AJC 3385	ANDES-A 1247	*Allobates ranoides*	Sabanalarga, Casanare	BSECO372-11	14	BOLD:ABA1656	KJ130698	KJ130662
AJC 3873	ANDES-A 1732	*Craugastor longirostris*	Sabana de Torres, Santander	BSECO302-11	41	BOLD:AAZ9403	KP149480	KP149268
AJC 3885	NA	*Craugastor longirostris*	Sabana de Torres, Santander	BSECO313-11	41	BOLD:AAZ9403	KP149315	KP149119
AJC 3881	ANDES-A 1733	*Dendrobates truncatus*	Sabana de Torres, Santander	BSECO310-11	42	BOLD:AAA5634	KP149428	KP149223
AJC 3882	ANDES-A 1734	*Dendrobates truncatus*	Sabana de Torres, Santander	BSECO311-11	42	BOLD:AAA5634	KP149285	KP149090
AJC 3886	ANDES-A 1735	*Dendrobates truncatus*	Sabana de Torres, Santander	BSECO314-11	42	BOLD:AAA5634	KP149287	KP149092
AJC 3502	ANDES-A 1222	*Dendropsophus ebraccatus*	San Vicente, Santander	BSAMS111-12	28	BOLD:ACC1363	KP149426	KP149221
AJC 3504	ANDES-A 1736	*Dendropsophus ebraccatus*	San Vicente, Santander	BSAMS113-12	28	BOLD:ACC1363	KP149355	KP149156
AJC 1740	ANDES-A 1593	*Dendropsophus mathiassoni*	San Juan de Arama, Meta	BSECO173-11	1	BOLD:ABA0401	KP149436	KP149230
AJC 1746	ANDES-A 1737	*Dendropsophus mathiassoni*	San Juan de Arama, Meta	BSECO179-11	1	BOLD:ABA0401	KP149479	KP149267
AJC 1749	ANDES-A 1738	*Dendropsophus mathiassoni*	San Juan de Arama, Meta	BSECO182-11	1	BOLD:ABA0401	KP149393	KP149189
AJC 2111	ANDES-A 1740	*Dendropsophus mathiassoni*	San Juan de Arama, Meta	BSECO217-11	1	BOLD:ABA0401	KP149304	KP149108
AJC 2112	ANDES-A 1741	*Dendropsophus mathiassoni*	San Juan de Arama, Meta	BSECO218-11	1	BOLD:ABA0401	KP149298	KP149102
AJC 2313	ANDES-A 1742	*Dendropsophus mathiassoni*	Orocué, Casanare	BSAMS083-12	1	BOLD:ABA0401	KP149389	KP149185
AJC 2322	ANDES-A 1163	*Dendropsophus mathiassoni*	Orocué, Casanare	BSAMS086-12	1	BOLD:ABA0401	KP149399	KP149214
AJC 2323	ANDES-A 1161	*Dendropsophus mathiassoni*	Orocué, Casanare	BSAMS087-12	1	Not sequenced for CO1	KP149399	Not sequenced for CO1
AJC 3923	ANDES-A 1270	*Dendropsophus mathiassoni*	San Juan de Arama, Meta	BSECO186-11	1	BOLD:ABA0401	KP149474	KP149262
AJC 3933	ANDES-A 1268	*Dendropsophus mathiassoni*	San Juan de Arama, Meta	BSECO196-11	1	BOLD:ABA0401	KP149309	KP149113
AJC 4052	ANDES-A 1085	*Dendropsophus mathiassoni*	Sabanalarga, Casanare	BSECO013-11	1	BOLD:ABA0401	KP149281	KP149086
AJC 4060	ANDES-A 1079	*Dendropsophus mathiassoni*	Sabanalarga, Casanare	BSECO008-11	1	BOLD:ABA0401	KP149305	KP149109
AJC 4070	ANDES-A 1066	*Dendropsophus mathiassoni*	Sabanalarga, Casanare	BSECO021-11	1	BOLD:ABA0401	KP149310	KP149114
AJC 4095	ANDES-A 1074	*Dendropsophus mathiassoni*	Sabanalarga, Casanare	BSECO046-11	1	BOLD:ABA0401	KP149464	KP149254
AJC 3869	NA	*Dendropsophus microcephalus*	Sabana de Torres, Santander	BSECO298-11	40	BOLD:AAC1019	KP149375	KP149173
AJC 3887	ANDES-A 1295	*Dendropsophus microcephalus*	Sabana de Torres, Santander	BSECO315-11	40	BOLD:AAC1019	KP149423	KP149218

AJC 4035	ANDES-A 1743	*Dendropsophus microcephalus*	San Vicente, Santander	BSAMS182-12	40	BOLD:AAC1019	KP149342	KP149145
AJC 4038	ANDES-A 1451	*Dendropsophus microcephalus*	San Vicente, Santander	BSAMS184-12	40	BOLD:AAC1019	KP149404	KP149199
AJC 3384	ANDES-A 1230	*Dendropsophus* cf. *stingi*	Sabanalarga, Casanare	BSECO371-11	15	BOLD:ABA0313	KP149313	KP149117
AJC 3999	ANDES-A 1491	*Dendropsophus* cf. *stingi*	Miraflores, Boyacá	BSECO152-11	15	BOLD:ABA0313	KJ817825	KJ817831
AJC 4001	ANDES-A 1466	*Dendropsophus* cf. *stingi*	Miraflores, Boyacá	BSECO154-11	15	BOLD:ABA0313	KP149441	KP149235
AJC 4003	ANDES-A 1492	*Dendropsophus* cf. *stingi*	Miraflores, Boyacá	BSECO156-11	15	BOLD:ABA0313	KJ817824	KJ817830
AJC 4004	ANDES-A 1493	*Dendropsophus* cf. *stingi*	Miraflores, Boyacá	BSECO157-11	15	BOLD:ABA0313	KJ817826	KJ817832
AJC 4005	ANDES-A 1467	*Dendropsophus* cf. *stingi*	Miraflores, Boyacá	BSECO158-11	15	BOLD:ABA0313	KP149369	KP149167
AJC 4110	ANDES-A 1130	*Dendropsophus* cf. *stingi*	Sabanalarga, Casanare	BSECO060-11	15	BOLD:ABA0313	KJ817827	KJ817833
AJC 4111	ANDES-A 1490	*Dendropsophus* cf. *stingi*	Sabanalarga, Casanare	BSECO061-11	15	BOLD:ABA0313	Pending	Pending
LSB 373	ANDES-A 1488	*Dendropsophus* cf. *stingi*	Pajarito, Boyacá	BSECO263-11	15	BOLD:ABA0313	KJ817828	KJ817834
LSB 374	ANDES-A 1489	*Dendropsophus* cf. *stingi*	Pajarito, Boyacá	BSECO264-11	15	BOLD:ABA0313	KJ817829	KJ817835
AJC 3837	ANDES-A 1277	*Dendropsophus subocularis*	Puente Nacional, Santander	BSECO276-11	36	Not sequenced for CO1	KP149434	Not sequenced for CO1
AJC 3845	ANDES-A 1278	*Dendropsophus subocularis*	Puente Nacional, Santander	BSECO284-11	36	BOLD:ABV9393	KP149306	KP149110
AJC 3847	ANDES-A 1274	*Dendropsophus subocularis*	Puente Nacional, Santander	BSECO286-11	36	BOLD:ABV9393	KP149412	KP149207
AJC 1742	ANDES-A 1744	*Elachistocleis ovalis*	San Juan de Arama, Meta	BSECO175-11	3	BOLD:ABA1508	KP149470	KP149259
AJC 2306	ANDES-A 1745	*Elachistocleis ovalis*	Orocué, Casanare	BSAMS082-12	3	BOLD:ABA1508	KP149445	KP149238
AJC 2381	ANDES-A 1157	*Elachistocleis ovalis*	Orocué, Casanare	BSAMS103-12	3	BOLD:ABA1508	KP149457	KP149247
AJC 3374	ANDES-A 1070	*Elachistocleis ovalis*	Sabanalarga, Casanare	BSECO091-11	3	BOLD:ABA1508	KP149484	KP149272
AJC 3377	ANDES-A 1069	*Elachistocleis ovalis*	Sabanalarga, Casanare	BSECO094-11	3	BOLD:ABA1508	KP149288	KP149093
AJC 3379	ANDES-A 1240	*Elachistocleis ovalis*	Sabanalarga, Casanare	BSECO096-11	3	BOLD:ABA1508	KP149400	KP149195
AJC 3970	ANDES-A 1239	*Elachistocleis ovalis*	Sabanalarga, Casanare	BSECO082-11	3	BOLD:ABA1508	KP149387	KP149184
AJC 3972	ANDES-A 1236	*Elachistocleis ovalis*	Sabanalarga, Casanare	BSECO084-11	3	BOLD:ABA1508	KP149487	KP149274
AJC 3507	ANDES-A 1032	*Engystomops pustulosus*	San Vicente, Santander	BSAMS116-12	29	BOLD:ABV7902	KP149280	KP149085
AJC 3859	ANDES-A 1746	*Engystomops pustulosus*	Piedecuesta, Santander	BSECO328-11	29	BOLD:ABV7902	KP149458	KP149248
AJC 3862	ANDES-A 1747	*Engystomops pustulosus*	Piedecuesta, Santander	BSECO331-11	29	BOLD:ABV7902	KP149413	KP149208
AJC 3872	ANDES-A 1748	*Engystomops pustulosus*	Sabana de Torres, Santander	BSECO301-11	29	BOLD:ABV7902	KP149356	KP149157
AJC 3875	ANDES-A 1749	*Engystomops pustulosus*	Sabana de Torres, Santander	BSECO304-11	29	BOLD:ABV7903	KP149340	KP149143
AJC 2302	ANDES-A 1750	*Espadarana andina*	Puente Nacional, Santander	BSECO122-11	7	BOLD:ABU6599	KP149354	KP149155
AJC 3387	ANDES-A 1751	*Espadarana andina*	Puente Nacional, Santander	BSECO105-11	7	BOLD:ABU6599	KP149447	KP149240
LSB 384	ANDES-A 1248	*Hyalinobatrachium esmeralda*	Pajarito, Boyacá	BSECO274-11	56	BOLD:ABU6258	KP149361	KP149161
LSB 369	ANDES-A 1818	*Hyloscirtus* cf. *phyllognathus*	Pajarito, Boyacá	BSECO259-11	54	BOLD:ABU6239	KP149337	KP149140
AJC 3443	ANDES-A 1752	*Hypsiboas boans*	San Juan de Arama, Meta	BSECO220-11	20	BOLD:AAI7867	KP149456	KP149246
AJC 3396	ANDES-A 1753	*Hypsiboas crepitans*	Puente Nacional, Santander	BSECO104-11	16	BOLD:ABZ3336	KP149283	KP149088
AJC 3505	ANDES-A 1528	*Hypsiboas crepitans*	San Vicente, Santander	BSAMS114-12	16	BOLD:ABZ3336	KP149290	KP149095
AJC 3838	ANDES-A 1529	*Hypsiboas crepitans*	Puente Nacional, Santander	BSECO277-11	16	BOLD:ABZ3336	KP149481	KP149269
AJC 3839	ANDES-A 1754	*Hypsiboas crepitans*	Puente Nacional, Santander	BSECO278-11	16	BOLD:ABA0429	KP149448	KP149241
AJC 3840	ANDES-A 1530	*Hypsiboas crepitans*	Puente Nacional, Santander	BSECO279-11	16	BOLD:ABZ3336	KP149420	KP149215
AJC 3842	ANDES-A 1531	*Hypsiboas crepitans*	Puente Nacional, Santander	BSECO281-11	16	BOLD:ABZ3336	KP149402	KP149197
AJC 3844	ANDES-A 1532	*Hypsiboas crepitans*	Puente Nacional, Santander	BSECO283-11	16	BOLD:ABZ3336	KP149316	KP149120
AJC 3925	ANDES-A 1534	*Hypsiboas crepitans*	San Juan de Arama, Meta	BSECO188-11	16	BOLD:ABA0429	KP149475	KP149263

AJC 3934	ANDES-A 1535	*Hypsiboas crepitans*	San Juan de Arama, Meta	BSECO197-11	16	BOLD:ABA0429	KP149477	KP149265
AJC 4010	ANDES-A 1507	*Hypsiboas crepitans*	Miraflores, Boyacá	BSECO370-11	16	BOLD:ABA0429	KP149317	KP149121
AJC 4014	ANDES-A 1508	*Hypsiboas crepitans*	Miraflores, Boyacá	BSECO163-11	16	BOLD:ABA0429	BSECO163	KP149256
AJC 4019	ANDES-A 1509	*Hypsiboas crepitans*	Miraflores, Boyacá	BSECO168-11	16	BOLD:ABA0429	KP149378	KP149176
AJC 4108	ANDES-A 1063	*Hypsiboas crepitans*	Sabanalarga, Casanare	BSECO058-11	16	BOLD:ABA0429	KP149370	KP149168
AJC 4116	ANDES-A 1284	*Hypsiboas crepitans*	Sabanalarga, Casanare	BSECO066-11	16	BOLD:ABA0429	KP149478	KP149266
AJC 3373	ANDES-A 1054	*Hypsiboas lanciformis*	Sabanalarga, Casanare	BSECO090-11	12	Not sequenced for CO1	KP149451	Pending
AJC 3971	ANDES-A 1231	*Hypsiboas lanciformis*	Sabanalarga, Casanare	BSECO083-11	12	Not sequenced for CO1	BSECO083	Pending
AJC 3973	ANDES-A 1053	*Hypsiboas lanciformis*	Sabanalarga, Casanare	BSECO085-11	12	Not sequenced for CO1	BSECO085	Not sequenced for CO1
AJC 3975	ANDES-A 1238	*Hypsiboas lanciformis*	Sabanalarga, Casanare	BSECO087-11	12	BOLD:ABX7699	KP149371	KP149169
AJC 3501	ANDES-A 1541	*Hypsiboas pugnax*	San Vicente, Santander	BSAMS110-12	27	BOLD:ABV8158	KP149407	KP149202
AJC 3503	ANDES-A 1207	*Hypsiboas pugnax*	San Vicente, Santander	BSAMS112-12	27	BOLD:ABV8158	KP149471	KP149169
AJC 3529	ANDES-A 1453	*Hypsiboas pugnax*	San Vicente, Santander	BSAMS124-12	27	BOLD:ABV8158	KP149296	KP149101
AJC 3870	ANDES-A 1533	*Hypsiboas pugnax*	Sabana de Torres, Santander	BSECO299-11	27	BOLD:ABV8158	KP149417	KP149212
AJC 3871	ANDES-A 1538	*Hypsiboas pugnax*	Sabana de Torres, Santander	BSECO300-11	27	BOLD:ABV8158	KP149418	KP149213
AJC 3874	ANDES-A 1539	*Hypsiboas pugnax*	Sabana de Torres, Santander	BSECO303-11	27	BOLD:ABV8158	KP149327	KP149130
AJC 3878	ANDES-A 1540	*Hypsiboas pugnax*	Sabana de Torres, Santander	BSECO307-11	27	BOLD:ABV8158	KP149409	KP149204
AJC 3921	ANDES-A 1755	*Hypsiboas punctatus*	San Juan de Arama, Meta	BSECO184-11	45	BOLD:ABA0585	KP149469	KP149258
AJC 3922	ANDES-A 1756	*Hypsiboas punctatus*	San Juan de Arama, Meta	BSECO185-11	45	BOLD:ABA0585	KP149390	KP149186
AJC 3928	ANDES-A 1757	*Hypsiboas punctatus*	San Juan de Arama, Meta	BSECO191-11	45	BOLD:ABA0585	KP149392	KP149188
AJC 3932	ANDES-A 1758	*Hypsiboas punctatus*	San Juan de Arama, Meta	BSECO195-11	45	BOLD:ABA0585	KP149476	KP149264
AJC 4064	ANDES-A 1044	*Hypsiboas punctatus*	Sabanalarga, Casanare	BSECO016-11	52	BOLD:ABA0584	KP149397	KP149193
AJC 4100	ANDES-A 1242	*Hypsiboas punctatus*	Sabanalarga, Casanare	BSECO051-11	52	BOLD:ABA0584	KP149307	KP149111
AJC 3848	ANDES-A 1761	*Leptodactylus colombiensis*	Puente Nacional, Santander	BSECO287-11	37	BOLD:ABV8030	KP149318	KP149122
AJC 3978	ANDES-A 1456	*Leptodactylus colombiensis*	Miraflores, Boyacá	BSECO132-11	37	BOLD:ABA1440	KP149468	KP149257
AJC 3981	ANDES-A 1460	*Leptodactylus colombiensis*	Miraflores, Boyacá	BSECO135-11	37	BOLD:ABA1440	KP149403	KP149198
AJC 3986	ANDES-A 1457	*Leptodactylus colombiensis*	Miraflores, Boyacá	BSECO140-11	37	BOLD:ABA1440	KP149444	KP149237
AJC 4000	ANDES-A 1762	*Leptodactylus colombiensis*	Miraflores, Boyacá	BSECO153-11	37	BOLD:ABA1440	KP149302	KP149106
LSB 378	ANDES-A 1250	*Leptodactylus colombiensis*	Pajarito, Boyacá	BSECO268-11	37	BOLD:ABA1440	KP149459	KP149249
AJC 2301	ANDES-A 1169	*Leptodactylus fuscus*	Orocué, Casanare	BSAMS081-12	6	BOLD:ABA0407	KP149398	KP149194
AJC 3467	ANDES-A 1763	*Leptodactylus fuscus*	San Juan de Arama, Meta	BSECO243-11	6	BOLD:ABA0407	KP149410	KP149205
AJC 3892	ANDES-A 1193	*Leptodactylus fuscus*	Sabana de Torres, Santander	BSECO319-11	6	BOLD:ABV8011	KP149449	KP149242
AJC 3893	ANDES-A 1188	*Leptodactylus fuscus*	Sabana de Torres, Santander	BSECO320-11	6	BOLD:ABV8011	KP149446	KP149239
AJC 4078	ANDES-A 1059	*Leptodactylus fuscus*	Sabanalarga, Casanare	BSECO029-11	6	BOLD:ABA0407	KP149301	KP149105
AJC 4088	ANDES-A 1048	*Leptodactylus fuscus*	Sabanalarga, Casanare	BSECO039-11	6	BOLD:ABA0407	KP149352	KP149153
AJC 4104	ANDES-A 1116	*Leptodactylus fuscus*	Sabanalarga, Casanare	BSECO054-11	6	Not sequenced for CO1	KP149349	Not sequenced for CO1
AJC 3509	ANDES-A 1760	*Leptodactylus insularum*	San Vicente, Santander	BSAMS117-12	30	BOLD:AAJ1153	KP149348	KP149150
AJC 3517	ANDES-A 1172	*Leptodactylus insularum*	San Vicente, Santander	BSAMS121-12	30	BOLD:AAJ1153	KP149424	KP149219
AJC 3429	ANDES-A 1145	*Leptodactylus knudseni*	Orocué, Casanare	BSAMS109-12	10	BOLD:ACA5872	KP149492	KP149277
AJC 3451	ANDES-A 1764	*Leptodactylus lineatus*	San Juan de Arama, Meta	BSECO228-11	22	BOLD:ABA0568	KP149432	KP149226
AJC 3456	ANDES-A 1765	*Leptodactylus lineatus*	San Juan de Arama, Meta	BSECO233-11	22	BOLD:ABA0568	KP149329	KP149132

AJC 3462	ANDES-A 1221	*Leptodactylus lineatus*	San Juan de Arama, Meta	BSECO238-11	22	BOLD:ABA0568	KP149293	KP149098
AJC 3448	ANDES-A 1759	*Leptodactylus mystaceus*	San Juan de Arama, Meta	BSECO225-11	21	BOLD:ABA0408	KP149336	KP149139
AJC 4092	ANDES-A 1109	*Leptodactylus mystaceus*	Sabanalarga, Casanare	BSECO043-11	21	BOLD:ABA0408	KP149359	KP149222
AJC 3453	ANDES-A 1212	*Phyllomedusa hypochondrialis*	San Juan de Arama, Meta	BSECO230-11	23	BOLD:ABA0575	KP149427	KP149222
AJC 3459	ANDES-A 1766	*Phyllomedusa hypochondrialis*	San Juan de Arama, Meta	BSECO252-11	23	BOLD:ABA0575	KP149279	KP149084
AJC 4072	ANDES-A 1103	*Phyllomedusa hypochondrialis*	Sabanalarga, Casanare	BSECO023-11	23	BOLD:ABA0575	KP149489	KP149275
AJC 4103	ANDES-A 1105	*Phyllomedusa hypochondrialis*	Sabanalarga, Casanare	BSECO053-11	23	BOLD:ABA0575	KP149374	KP149172
AJC 2316	ANDES-A 1353	*Physalaemus fischeri*	Orocué, Casanare	BSAMS084-12	8	BOLD:ABA1319	KP149433	KP149227
AJC 3316	ANDES-A 1162	*Physalaemus fischeri*	Orocué, Casanare	BSAMS105-12	8	BOLD:ABA1319	Pending	KP149134
AJC 3452	ANDES-A 1292	*Physalaemus fischeri*	San Juan de Arama, Meta	BSECO229-11	8	BOLD:ABA1319	KP149472	KP149261
AJC 3956	ANDES-A 1767	*Physalaemus fischeri*	San Juan de Arama, Meta	BSECO254-11	8	BOLD:ABA1319	KP149364	KP149163
AJC 4065	ANDES-A 1104	*Physalaemus fischeri*	Sabanalarga, Casanare	BSECO017-11	8	BOLD:ABA1319	KP149328	KP149131
AJC 4090	ANDES-A 1097	*Physalaemus fischeri*	Sabanalarga, Casanare	BSECO041-11	8	BOLD:ABA1319	KP149373	KP149171
AJC 3894	ANDES-A 1768	*Pleurodema brachyops*	Sabana de Torres, Santander	BSECO321-11	43	BOLD:AAE4476	KP149314	KP149118
LSB 385	ANDES-A 1254	*Pristimantis carranguerorum*	Pajarito, Boyacá	BSECO275-11	57	BOLD:ABU7033	KP149324	KP149128
AJC 3472	ANDES-A 1778	*Pristimantis douglasi*	El Rasgón, Santander	BSECO343-11	25	BOLD:ABV7786	KP149380	KP149178
AJC 3486	ANDES-A 1769	*Pristimantis douglasi*	El Rasgón, Santander	BSECO355-11	25	BOLD:ABV7786	KP149289	KP149094
AJC 3492	ANDES-A 1770	*Pristimantis douglasi*	El Rasgón, Santander	BSECO361-11	25	BOLD:ABV7786	KP149286	KP149091
AJC 3977	ANDES-A 1518	*Pristimantis frater*	Miraflores, Boyacá	BSECO131-11	47	BOLD:ABA0337	KP149367	KP149165
AJC 3982	ANDES-A 1520	*Pristimantis frater*	San Juan de Arama, Meta	BSECO136-11	47	BOLD:ABA0337	KP149368	KP149166
AJC 3984	ANDES-A 1522	*Pristimantis frater*	Miraflores, Boyacá	BSECO138-11	47	BOLD:ABA0337	KP149415	KP149210
AJC 3987	ANDES-A 1523	*Pristimantis frater*	Miraflores, Boyacá	BSECO141-11	47	BOLD:ABA0337	KP149363	KP149162
AJC 4011	ANDES-A 1499	*Pristimantis frater*	Miraflores, Boyacá	BSECO160-11	47	BOLD:ABA0337	KP149326	KP149129
AJC 4015	ANDES-A 1501	*Pristimantis frater*	Miraflores, Boyacá	BSECO164-11	47	BOLD:ABA0337	KP149461	KP149251
AJC 4018	ANDES-A 1503	*Pristimantis frater*	Miraflores, Boyacá	BSECO167-11	47	BOLD:ABA0337	KP149414	KP149209
AJC 3490	ANDES-A 1771	*Pristimantis lutitus*	El Rasgón, Santander	BSECO359-11	26	BOLD:ABV9411	KP149401	KP149196
AJC 3407	NA	*Pristimantis miyatai*	PuenteNacionalSAN	BSECO118-11	18	Not sequenced for CO1	KP149388	Not sequenced for CO1
AJC 3475	ANDES-A 1772	*Pristimantis miyatai*	El Rasgón, Santander	BSECO345-11	18	BOLD:AAT9778	KP149490	KP149276
AJC 3477	ANDES-A 1773	*Pristimantis miyatai*	El Rasgón, Santander	BSECO346-11	18	BOLD:AAT9778	KP149395	KP149191
AJC 3478	ANDES-A 1779	*Pristimantis miyatai*	El Rasgón, Santander	BSECO347-11	18	BOLD:AAT9778	KP149440	KP149234
AJC 3479	ANDES-A 1774	*Pristimantis miyatai*	El Rasgón, Santander	BSECO348-11	18	BOLD:AAT9778	KP149396	KP149192
AJC 3498	ANDES-A 1775	*Pristimantis miyatai*	El Rasgón, Santander	BSECO367-11	18	BOLD:AAT9778	KP149437	KP149231
AJC 3499	ANDES-A 1776	*Pristimantis miyatai*	El Rasgón, Santander	BSECO368-11	18	BOLD:AAT9778	KP149300	KP149104
AJC 3856	ANDES-A 1777	*Pristimantis miyatai*	Puente Nacional, Santander	BSECO295-11	18	BOLD:ABV8595	KP149351	KP149152
AJC 3995	ANDES-A 1497	*Pristimantis savagei*	Miraflores, Boyacá	BSECO148-11	49	BOLD:AAV6745	KP149382	KP149180
AJC 4012	ANDES-A 1500	*Pristimantis savagei*	Miraflores, Boyacá	BSECO161-11	49	BOLD:AAV6745	KP149291	KP149096
LSB 375	ANDES-A 1260	*Pristimantis savagei*	Pajarito, Boyacá	BSECO265-11	49	BOLD:AAV6745	KP149299	KP149103
LSB 377	ANDES-A 1259	*Pristimantis savagei*	Pajarito, Boyacá	BSECO267-11	49	BOLD:AAV6745	KP149405	KP149200
LSB 381	ANDES-A 1256	*Pristimantis savagei*	Pajarito, Boyacá	BSECO271-11	49	BOLD:AAV6745	KP149425	KP149220
LSB 383	ANDES-A 1255	*Pristimantis savagei*	Pajarito, Boyacá	BSECO273-11	49	BOLD:AAV6745	KP149411	KP149206
AJC 3861	ANDES-A 1782	*Pristimantis taeniatus*	Piedecuesta, Santander	BSECO330-11	39	BOLD:AAT9777	KP149311	KP149115

AJC 3864	ANDES-A 1780	*Pristimantis taeniatus*	Piedecuesta, Santander	BSECO333-11	39	BOLD:AAT9777	KP149358	KP149159
AJC 3867	ANDES-A 1781	*Pristimantis taeniatus*	Piedecuesta, Santander	BSECO336-11	39	BOLD:AAT9777	KP149365	KP149164
AJC 2113	ANDES-A 1783	*Pristimantis vilarsi*	San Juan de Arama, Meta	BSECO219-11	5	BOLD:ABA0338	KP149391	KP149187
AJC 3450	ANDES-A 1286	*Pristimantis vilarsi*	San Juan de Arama, Meta	BSECO227-11	5	BOLD:ABA0338	KP149334	KP149138
AJC 3944	ANDES-A 1784	*Pristimantis vilarsi*	San Juan de Arama, Meta	BSECO207-11	5	BOLD:ABA0338	KP149278	KP149083
AJC 3945	ANDES-A 1785	*Pristimantis vilarsi*	San Juan de Arama, Meta	BSECO208-11	5	BOLD:ABA0338	KP149438	KP149232
AJC 3946	ANDES-A 1786	*Pristimantis vilarsi*	San Juan de Arama, Meta	BSECO209-11	5	BOLD:ABA0338	KP149384	KP149181
AJC 3957	ANDES-A 1787	*Pristimantis vilarsi*	San Juan de Arama, Meta	BSECO249-11	5	BOLD:ABA0338	KP149333	KP149137
AJC 2382	ANDES-A 1121	*Pseudis paradoxa*	Orocué, Casanare	BSAMS104-12	11	BOLD:ACA5628	KP149394	KP149190
AJC 3454	ANDES-A 1227	*Pseudopaludicola llanera*	San Juan de Arama, Meta	BSECO231-11	24	BOLD:ABA1351	KP149338	KP149141
AJC 3799	ANDES-A 1789	*Pseudopaludicola llanera*	San Juan de Arama, Meta	BSECO245-11	24	BOLD:ABA1350	KP149453	KP149244
AJC 4109	ANDES-A 1117	*Pseudopaludicola llanera*	Sabanalarga, Casanare	BSECO059-11	24	BOLD:ABA1350	KP149292	KP149097
AJC 4115	ANDES-A 1253	*Pseudopaludicola llanera*	Sabanalarga, Casanare	BSECO065-11	24	BOLD:ABA1350	KP149482	KP149270
AJC 4117	ANDES-A 1115	*Pseudopaludicola llanera*	Sabanalarga, Casanare	BSECO067-11	24	BOLD:ABA1350	KP149332	KP149136
AJC 4127	ANDES-A 1114	*Pseudopaludicola llanera*	Sabanalarga, Casanare	BSECO078-11	24	BOLD:ABA1350	KP149483	KP149271
AJC 4037	ANDES-A 1790	*Pseudopaludicola pusilla*	San Vicente, Santander	BSAMS183-12	50	BOLD:ACC1655	KP149344	KP149147
AJC 4039	ANDES-A 1791	*Pseudopaludicola pusilla*	San Vicente, Santander	BSAMS185-12	50	BOLD:ACC1655	KP149486	KP149273
AJC 3398	ANDES-A 1476	*Rheobates palmatus*	Puente Nacional, Santander	BSECO130-11	17	BOLD:AAY0187	KJ130701	KJ130665
AJC 3401	ANDES-A 1477	*Rheobates palmatus*	Puente Nacional, Santander	BSECO111-11	17	BOLD:AAY0187	KJ130725	KJ130691
AJC 3403	ANDES-A 1478	*Rheobates palmatus*	Puente Nacional, Santander	BSECO110-11	17	BOLD:AAY0187	KJ130724	KJ130690
AJC 3404	ANDES-A 1479	*Rheobates palmatus*	Puente Nacional, Santander	BSECO119-11	17	BOLD:AAY0187	KJ130711	KJ130676
AJC 3406	ANDES-A 1480	*Rheobates palmatus*	Puente Nacional, Santander	BSECO113-11	17	BOLD:AAY0187	KJ130721	KJ130687
AJC 3514	ANDES-A 603	*Rheobates palmatus*	San Vicente, Santander	BSAMS119-12	17	Not sequenced for CO1	KJ130706	Not sequenced for CO1
AJC 3526	ANDES-A 1481	*Rheobates palmatus*	San Vicente, Santander	BSAMS123-12	17	BOLD:ACC1597	KJ130702	KJ130666
AJC 3860	ANDES-A 1482	*Rheobates palmatus*	Piedecuesta, Santander	BSECO329-11	17	BOLD:ABV8088	KJ130699	KJ130663
AJC 3953	ANDES-A 1483	*Rheobates palmatus*	Puente Nacional, Santander	BSECO128-11	17	BOLD:AAY0187	KJ130700	KJ130664
AJC 3954	ANDES-A 1484	*Rheobates palmatus*	Puente Nacional, Santander	BSECO129-11	17	BOLD:AAY0187	KJ130705	Pending
AJC 3533	ANDES-A 1213	*Rhinella humboldti*	San Vicente, Santander	BSAMS126-12	34	BOLD:ACC1400	KP149421	KP149216
AJC 3968	ANDES-A 1038	*Rhinella humboldti*	Sabanalarga, Casanare	BSECO080-11	34	Not sequenced for CO1	KP149346	Not sequenced for CO1
AJC 4023	ANDES-A 1792	*Rhinella humboldti*	San Juan de Arama, Meta	BSECO257-11	34	Not sequenced for CO1	KP149473	Not sequenced for CO1
AJC 4024	ANDES-A 1793	*Rhinella humboldti*	San Juan de Arama, Meta	BSECO253-11	34	Not sequenced for CO1	KP149366	Not sequenced for CO1
AJC 4073	ANDES-A 1034	*Rhinella humboldti*	Sabanalarga, Casanare	BSECO024-11	34	Not sequenced for CO1	KP149488	Not sequenced for CO1
AJC 3991	ANDES-A 1465	*Rhinella margaritifera*	Miraflores, Boyacá	BSECO145-11	48	BOLD:ABU5671	KP149312	KP149116
AJC 3994	ANDES-A 1464	*Rhinella margaritifera*	Miraflores, Boyacá	BSECO147-11	48	BOLD:ABU5671	KP149339	KP149142
AJC 3996	ANDES-A 1463	*Rhinella margaritifera*	Miraflores, Boyacá	BSECO149-11	48	BOLD:ABU5671	KP149416	KP149211
LSB 368	ANDES-A 1817	*Rhinella margaritifera*	Pajarito, Boyacá	BSECO258-11	48	BOLD:ABU5671	KP149322	KP149126
LSB 380	ANDES-A 1261	*Rhinella margaritifera*	Pajarito, Boyacá	BSECO270-11	48	BOLD:ABU5671	KP149282	KP149087
AJC 3850	ANDES-A 1794	*Rhinella marina*	Puente Nacional, Santander	BSECO289-11	38	BOLD:AAB1186	KP149353	KP149154
AJC 3851	ANDES-A 1795	*Rhinella marina*	Puente Nacional, Santander	BSECO290-11	38	BOLD:AAB1186	KP149350	KP149151
AJC 3852	ANDES-A 1796	*Rhinella marina*	Puente Nacional, Santander	BSECO291-11	38	BOLD:AAB1186	KP149422	KP149217
AJC 3877	ANDES-A 1797	*Rhinella marina*	Sabana de Torres, Santander	BSECO306-11	38	BOLD:AAB1186	KP149357	KP149158

AJC 3895	ANDES-A 1798	*Rhinella marina*	Sabana de Torres, Santander	BSECO322-11	38	BOLD:AAB1186	KP149331	KP149135
AJC 3943	ANDES-A 1799	*Rhinella marina*	San Juan de Arama, Meta	BSECO206-11	38	Not sequenced for CO1	KP149485	Not sequenced for CO1
AJC 3997	ANDES-A 1800	*Rhinella marina*	Miraflores, Boyacá	BSECO151-11	38	Not sequenced for CO1	KP149383	Not sequenced for CO1
AJC 4002	ANDES-A 1801	*Rhinella marina*	Miraflores, Boyacá	BSECO155-11	38	Not sequenced for CO1	KP149360	Not sequenced for CO1
AJC 4020	ANDES-A 1802	*Rhinella marina*	Miraflores, Boyacá	BSECO169-11	38	Not sequenced for CO1	KP149450	Not sequenced for CO1
AJC 4021	ANDES-A 1803	*Rhinella marina*	Miraflores, Boyacá	BSECO170-11	38	Not sequenced for CO1	KP149297	Not sequenced for CO1
AJC 4029	ANDES-A 1804	*Rhinella marina*	San Juan de Arama, Meta	BSECO256-11	38	Not sequenced for CO1	KP149430	Not sequenced for CO1
AJC 4077	ANDES-A 1805	*Rhinella marina*	Sabanalarga, Casanare	BSECO028-11	38	Not sequenced for CO1	KP149335	Not sequenced for CO1
AJC 4125	ANDES-A 1806	*Rhinella marina*	Sabanalarga, Casanare	BSECO075-11	38	Not sequenced for CO1	KP149325	Not sequenced for CO1
LSB 376	NA	*Rulyrana flavopunctata*	Pajarito, Boyacá	BSECO266-11	55	BOLD:ABU6070	KP149462	KP149252
AJC 1747	ANDES-A 1808	*Scinax* cf. *kennedyi*	San Juan de Arama, Meta	BSECO180-11	2	BOLD:ABA0510	KP149308	KP149112
AJC 4051	ANDES-A 1062	*Scinax* cf. *kennedyi*	Sabanalarga, Casanare	BSECO012-11	2	Not sequenced for CO1	KP149321	Not sequenced for CO1
AJC 4061	ANDES-A 1058	*Scinax* cf. *kennedyi*	Sabanalarga, Casanare	BSECO009-11	2	BOLD:ABA0510	KP149429	KP149224
AJC 4074	ANDES-A 1060	*Scinax* cf. *kennedyi*	Sabanalarga, Casanare	BSECO025-11	2	BOLD:ABA0510	KP149463	KP149253
AJC 1741	ANDES-A 1807	*Scinax* cf. *kennedyi*	San Juan de Arama, Meta	BSECO174-11	2	BOLD:ABA0510	KP149408	KP149203
AJC 3422	ANDES-A 599	*Scinax rostratus*	San Vicente, Santander	BSAMS108-12	19	BOLD:ACC1535	KP149284	KP149089
AJC 3506	ANDES-A 1809	*Scinax rostratus*	San Vicente, Santander	BSAMS115-12	19	BOLD:ACC1535	KP149435	KP149229
AJC 2324	ANDES-A 1165	*Scinax ruber*	Orocué, Casanare	BSAMS088-12	9	Not sequenced for CO1	KP149491	Not sequenced for CO1
AJC 3378	ANDES-A 1504	*Scinax ruber*	Sabanalarga, Casanare	BSECO095-11	13	BOLD:ABA0385	KP149452	KP149243
AJC 3446	ANDES-A 1290	*Scinax ruber*	San Juan de Arama, Meta	BSECO223-11	13	BOLD:ABA0385	KP149466	KP149255
AJC 3532	ANDES-A 1210	*Scinax ruber*	San Vicente, Santander	BSAMS125-12	33	BOLD:ACC1578	KP149347	KP149149
AJC 3534	ANDES-A 1449	*Scinax ruber*	San Vicente, Santander	BSAMS127-12	33	BOLD:ACC1578	KP149295	KP149100
AJC 3884	ANDES-A 1810	*Scinax ruber*	Sabana de Torres, Santander	BSECO312-11	9	BOLD:ABV8597	KP149330	KP149133
AJC 3936	ANDES-A 1811	*Scinax ruber*	San Juan de Arama, Meta	BSECO199-11	13	BOLD:ABA0385	KP149294	KP149099
AJC 3940	ANDES-A 1812	*Scinax ruber*	San Juan de Arama, Meta	BSECO203-11	13	BOLD:ABA0385	KP149320	KP149124
AJC 4053	ANDES-A 1040	*Scinax ruber*	Sabanalarga, Casanare	BSECO014-11	13	BOLD:ABA0385	KP149379	KP149177
AJC 4054	ANDES-A 1046	*Scinax ruber*	Sabanalarga, Casanare	BSECO015-11	13	BOLD:ABA0385	KP149442	KP149236
AJC 1743	ANDES-A 1813	*Scinax wandae*	San Juan de Arama, Meta	BSECO176-11	4	BOLD:ABA0452	KP149376	KP149174
AJC 3461	ANDES-A 1287	*Scinax wandae*	San Juan de Arama, Meta	BSECO237-11	4	BOLD:ABA0452	KP149431	KP149225
AJC 3464	ANDES-A 1814	*Scinax wandae*	San Juan de Arama, Meta	BSECO240-11	4	BOLD:ABA0452	KP149460	KP149250
AJC 3942	ANDES-A 1815	*Scinax wandae*	San Juan de Arama, Meta	BSECO205-11	4	BOLD:ABA0452	KP149372	KP149170
AJC 3974	ANDES-A 1072	*Scinax wandae*	Sabanalarga, Casanare	BSECO086-11	46	BOLD:ABA1339	KP149323	KP149127
AJC 4105	ANDES-A 1077	*Scinax wandae*	Sabanalarga, Casanare	BSECO056-11	46	BOLD:ABA1339	KP149381	KP149179
AJC 4120	ANDES-A 1234	*Scinax wandae*	Sabanalarga, Casanare	BSECO070-11	46	BOLD:ABA1339	KP149319	KP149123
AJC 3525	ANDES-A 1816	*Smilisca phaeota*	San Vicente, Santander	BSAMS122-12	32	BOLD:AAB3398	KP149385	KP149182
AJC 4063	ANDES-A 1035	*Trachycephalus thyphonius*	Sabanalarga, Casanare	BSECO011-11	51	BOLD:ABA1587	KP149406	KP149201
